# Modeling the Effect on a Novel Fungal Peptaibol Placed in an All-Atom Bacterial Membrane Mimicking System via Accelerated Molecular Dynamics Simulations

**DOI:** 10.3390/life13122288

**Published:** 2023-11-30

**Authors:** Chetna Tyagi, Tamás Marik, András Szekeres, Csaba Vágvölgyi, László Kredics, Ferenc Ötvös

**Affiliations:** 1 Department of Microbiology, Faculty of Science and Informatics, University of Szeged, Közép fasor 52, H-6726 Szeged, Hungary; mariktamas88@gmail.com (T.M.); szandras@bio.u-szeged.hu (A.S.); csaba@bio.u-szeged.hu (C.V.); kredics@bio.u-szeged.hu (L.K.); 2 Institute of Biochemistry, Biological Research Centre, Temesvári krt. 62, H-6726 Szeged, Hungary; otvos@brc.hu

**Keywords:** peptaibol, bilayer membrane, *Trichoderma*, molecular dynamics simulations

## Abstract

We previously reported on a novel peptaibol, named Tripleurin XIIc (TPN), an 18-residue long sequence produced by the fungus *Trichoderma pleuroti*. We elucidated its 3D structure via classical and accelerated molecular dynamics simulation (aMD) methods and reported the folding dynamics of TPN in water and chloroform solvents. Peptaibols, in general, are insoluble in water, as they are amphipathic and may prefer hydrophobic environments like transmembrane regions. In this study, we attempted to use aMD simulations to model an all-atom bacterial membrane system while placing a TPN molecule in its vicinity. The results highlighted that TPN was able to introduce some disorder into the membrane and caused lipid clustering. It could also enter the transmembrane region from the water-bilayer interface. The structural dynamics of TPN in the transmembrane region revealed a single energetically stable conformation similar to the one obtained from water and chloroform solvent simulations reported by us previously. However, this linear structure was found to be at the local energy minimum (stable) in water but at a metastable intermediate state (higher energy) in chloroform. Therefore, it could be said that the water solvent can be successfully used for folding simulations of peptaibols.

## 1. Introduction

Peptaibols are linear, non-ribosomally produced amphipathic polypeptides of fungal origin, mostly comprising a high ratio of unusual amino acids. Non-standard amino acid residues like α-aminoisobutyric acid (Aib), hydroxyproline (Hyp), and D-isovaline (Div), and C-terminal alcohol residues like phenylalaninol (Pheol), valinol (Valol), etc., along with an acetylated N-terminus (Ac), are characteristic for these peptides ranging 7–20 amino acid residues in length [1,2,3]. They are synthesized by large modular enzymes called non-ribosomal peptide synthetases (NRPSs), where a single module contains multiple catalytic domains responsible for the incorporation of a single amino acid residue into the peptide chain [4,5,6]. Tripleurins are a newly identified group of peptaibols produced by the fungus *Trichoderma pleuroti*, which causes green mold disease in oyster mushroom cultivation (*Pleurotus ostreatus*) [7]. They have also been reported to be potential growth inhibitors of oyster mushroom mycelia. In a previous work, we carried out aMD simulations to elucidate the three-dimensional structure of the most produced Tripleurin sequence, namely, Tripleurin XIIc or TPN XIIc. The simulations were carried out in water and chloroform as solvents to compare the folding dynamics of TPN in hydrophilic (water) and hydrophobic (chloroform) environments [8]. We observed that two distinct structural clusters are formed in water: one with an unfolded C-terminus and a bent backbone and the other with a folded C-terminus (3_10_ helix) and linear backbone. Similarly, two distinct clusters were also obtained in chloroform: one with a folded, linear backbone and the other with a folded, curved backbone that resembled a hairpin-like structure. Surprisingly, the hairpin-like structure corresponds to the lowest energy conformation in chloroform, while the linear structure was obtained at a higher energy level [8].

It has been reported during experimental setups that peptaibols are insoluble in water. However, in reality, they must exist in some way at the water-bilayer interface to be transported from the producer fungus to a host membrane. This suggests the need to carry out simulations in water and a more hydrophobic solvent. For these reasons, we planned to place folded conformations of the TPN peptide at the water-bilayer interface and run advanced aMD simulations.

**Figure 1:**
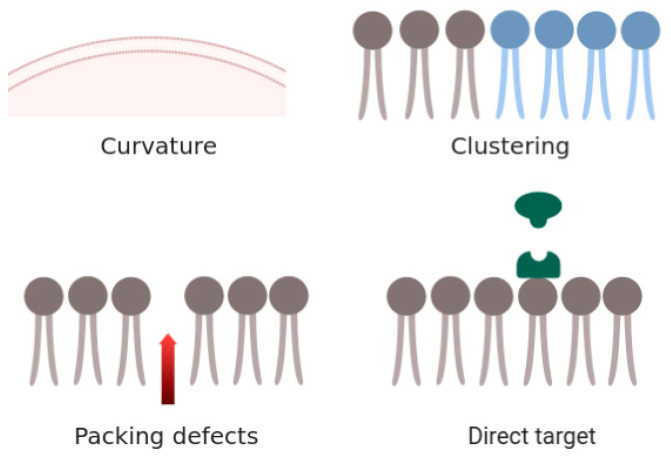
Various mechanisms by which antimicrobial compounds may target lipid membranes (adapted from Epand et al. [19]).

Bioactive peptides owe their activity to their membrane perturbation abilities. The most common mechanism of targeting a cell membrane by antimicrobial agents is through altering its bulk properties [9,10,11], such as membrane curvature [12,13,14,15], lipid clustering [16,17,18], packing defects [19], or direct targeting [20,21] (Figure 1). The membrane curvature and the intrinsic curvature of each monolayer of the cell membrane have been associated with the action of many antimicrobial peptides in terms of modulating their potency. The ability to introduce curvature by short peptides may even be correlated with the introduction of membrane thinning when the peptide is in a transmembrane orientation, as in the case of trichovirin-XII [22].

Another mechanism is by the clustering of lipid components, as in the case of anionic lipid clustering, due to the presence of cationic peptides. It has also been observed that more hydrophobic sequences preferentially form oligomeric transmembrane alignments (i.e., formation of ion channels or pores) as in the case of Alamethicin, while charged amphipathic sequences remain at the membrane surface and cause disruption of the phospholipid chain packing [23,24]. Such lipid clustering can introduce boundary defects and disrupt natural domains in the bilayer membrane. Packing defects in the bilayer membrane may also result in the loss of the permeability barrier. Meanwhile, the last mechanism, that of “direct action” against specific lipids of the membrane, remains the most important avenue for rational antimicrobial discovery. By rational, we mean that an antimicrobial agent must be designed to have the highest efficacy against the targeted bacteria while being the least toxic to the human host. The agents targeting membranes must target those lipid species found in abundance in bacterial membranes while absent or meagerly present in mammalian membranes. For example, anionic lipids such as phosphatidylglycerol (PG) and zwitterionic lipids such as phosphatidylethanolamine (PE) are mainly exposed on the outer surface of bacterial membranes, while they are found on the cytoplasmic surface of eukaryotic membranes [19]. In membrane mixtures of PG and PE, an antimicrobial agent may cause segregated clustering, as the anionic lipids preferentially bind to the cationic peptide. The main role of PE in bacterial membranes is to spread out the negative charge, assemble membrane proteins, and help the membrane proteins to correctly fold. Therefore, the clustering of membrane phospholipids results in considerable consequences, which must be discussed as a potential therapeutic avenue.

We created a simplistic bacterial membrane system containing a mixture of PE and PG lipids with a ratio of 3:1 to observe the effect of TPN peptide on various membrane properties. aMD is a state-of-the-art simulation technique that speeds up the process of sampling the potential energy surface within shorter simulation times. We created an all-atom representation for this system and, in this work, discuss the various observations made during this experiment.

## 2. Methodology

The primary structure of TPN is as follows:

AcAib1-Ser2-Ala3-Aib4-Vxx5-Gln6-Vxx7-Aib8-Vxx9-Ala10-Vxx11-Aib12-Pro13-Lxx14-Aib15-Vxx16-Gln17-Pheol18

The ambiguous residue positions were predicted based on the sequence of non-ribosomal peptide synthetase (NRPS) proteins using the antiSMASH database server [25]. Positions Vxx5, Vxx7, and Vxx11 were predicted to be D-isovalines, Vxx9 and Vxx16 were predicted to be valines, while Lxx14 was predicted as leucine. This prediction adds a large number of D-amino acid residues, which may have a strong influence on the overall structure and bioactivity.

We prepared a simplistic representation of a bacterial membrane system using the Packmol-Memgen [26] utility available through AmberTools18 [27]. The two lipid components 1,2-dioleoyl-sn-glycero-3-phosphoethanolamine (DOPE) and 1,2-dioleoyl-sn-glycero-3-[phospho-rac-(1-glycerol)] (DOPG) were added with a ratio of 3:1. A fully-folded conformation of TPN from our previous experiments was placed above this membrane patch. Water was represented by the TIP3P model and 5448 water residues were added along with 20 potassium ions (K^+^) to neutralize the system with a total length in the Z coordinate of 110 Å. The final system was energy-minimized using the steepest descent algorithm for the first 5,000 cycles, then switched to the conjugate gradient algorithm for 10,000 cycles. After that, two heating steps were carried out: first, to 100 K for 25,000 steps and then, from 100 K to 303 K for 50,000 steps. The temperature scaling was carried out using the Langevin thermostat, while the pressure was regulated using the default Berendsen barostat for all corresponding calculations. SHAKE bond length constraints were applied to all bonds involving hydrogen. The system was equilibrated in 10 steps of 500 ps each. A short classical MD run was also carried out for 125 ns to obtain average dihedral and potential energies (kcal mol^−1^) at a temperature of 300 K. Periodic boundary conditions with constant pressure were used using a Berendsen barostat in each case.

The aMD simulation was carried out in steps for a total of 2200 ns at a temperature of 300 K with a 2 fs time step. The electrostatic interactions were calculated using particle mesh Ewald summation (PME) [28] and long-range interactions were also calculated with a cutoff of 10 Angstroms. The temperature scaling was carried out using the Langevin thermostat without pressure scaling during aMD. Energies and boost information were written at every 1000 steps. The GPU machines available through the NIIF High-Performance Computing supercomputer at the University of Debrecen on the partition prod-gpu-k40-Leo nodes with 3 × Nvidia K40X CUDA8 were utilized for all aMD simulations. All simulations were carried out using the pmemd.cuda implementation of Amber14, also available at the cluster. aMD can be carried out using three criteria: (i) independently boosting the torsional terms of the potential (iamd = 2) or (ii) the whole potential at once (iamd = 1), and (iii) boosting the whole potential with an extra boost to torsions (iamd = 3).

The dihedral-based PCA was carried out using the cpptraj module. MD trajectories were analyzed by the program ‘grcarma’ [29,30] to generate the highest populated clusters using the top three principal components (PC) calculated from dihedral PCA and to write their representative structures in pdb format files. For dihedral PCA, the phi-psi torsion angles were calculated for all amino acid residues and the covariance matrix was calculated. The eigenvectors were calculated based on the covariance matrix.

An important aspect of an aMD calculation is to reweight the distribution to remove the effect of the boost applied to the system and to recover the original free energy landscapes. To recover this distribution, ‘amd.log’ is generated during the run, which contains information about the extra boost added to each snapshot of the trajectory. Theoretically, aMD simulations can be reweighted by the Boltzmann factors of the corresponding boost potential (i.e., eΔV/kBT) and averaged over each bin of selected reaction coordinate(s) to obtain the canonical ensemble, a technique called the exponential average which, however, suffers from large statistical noise especially if higher boost was applied. To overcome this problem, a method called Maclaurin series expansion was used, which approximates the exponential Boltzmann factor and significantly reduces the energetic noise [31]. The first two principal components are reweighted using the Maclaurin series expansion method in this study.

The lipid order parameters of the acyl chains were also determined, which can be directly compared with the experimental S_CD_ values. S_CD_ is a measure of the relative orientation of the C-D bonds with respect to bilayer normal and can be calculated as |S_CD_| = 0.5 <3cos2θ − 1>, where θ is the angle between bilayer normal and the vector joining Ci to its deuterium atom, where <> means the average of all lipid molecules. All contributions from conformational disorder, local tilting known as lipid wobble, and collective motions constitute the S_CD_ parameter and thus, can be a measure of membrane fluidity [32,33].

## 3. Results

**Figure 2:**
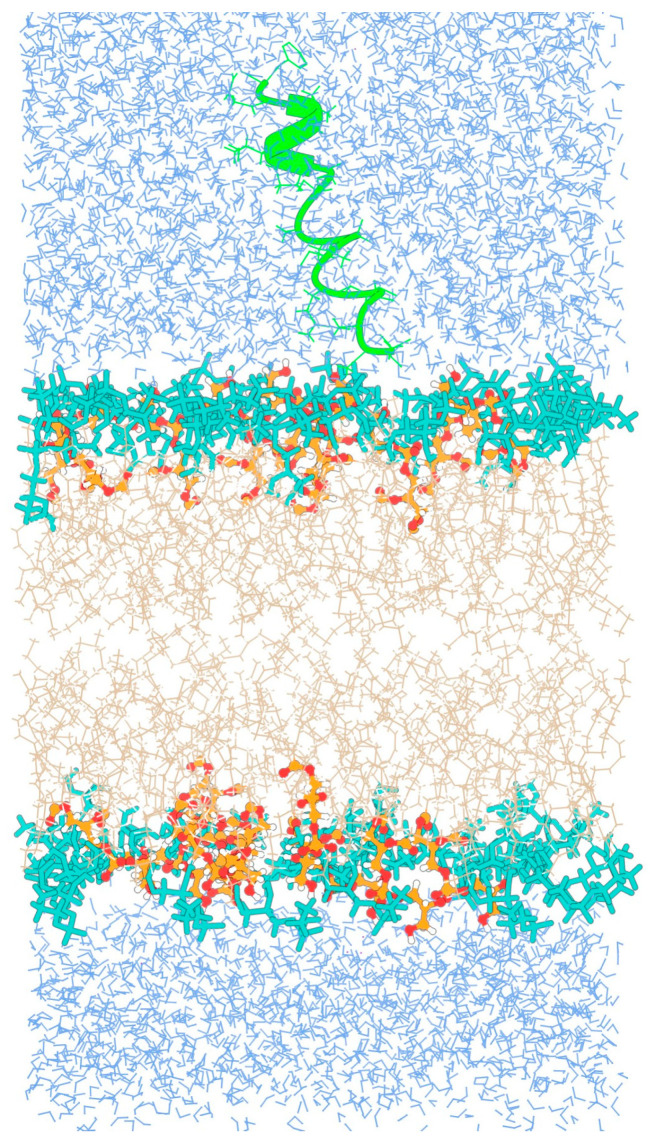
Initial configuration of the folded TPN peptide with respect to the bilayer membrane. PG lipid heads (orange), PE lipid heads (dark cyan), OL lipid chains (beige), TPN (green).

The fully folded coil-like conformation of TPN was taken from our previously published work [8]. We reported earlier that the TPN XIIc peptide shows a higher propensity for a spiral-like helix at the N-terminal and α/3_10_ helix at the C-terminal with a slight backbone bend in water solvent, and for a γ-turn in the central region in chloroform solvent that may induce backbone reversal. The C-terminal mostly folds into a 3_10_ helix in both solvents. This work is an extension of our previous results in which we had performed extended aMD simulations of TPN while placed inside water and chloroform solvents. The dynamics of TPN were studied in detail by PCA using internal/dihedral angle coordinates, and the trajectory was clustered by dihedral angle PCA. We chose the most likely folded conformation marked as cluster 8 in water solvent or as cluster 1 in chloroform and placed it above the DOPE:DOPG membrane patch (Figure 2) for this study. The H atoms were stripped from the water molecules to reduce the overall size of the trajectory for easier analysis.

### 3.1. Behavior of TPN XIIc Peptide in DOPE:DOPG System with 3:1 Ratio

**Figure 3:**
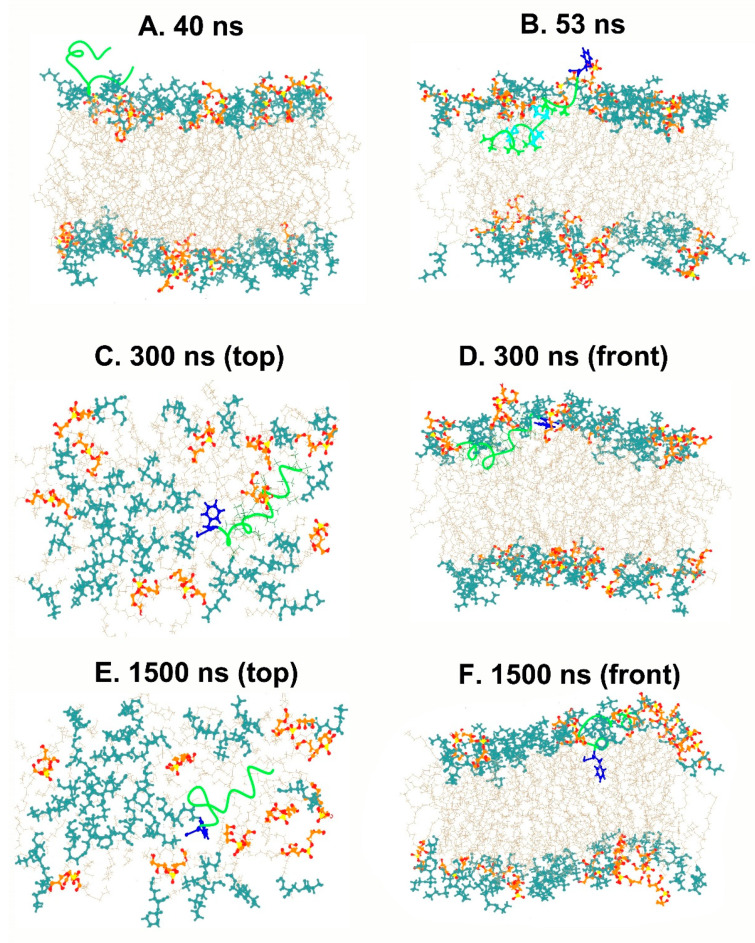
Snapshots from the simulation at different time steps showing the movement of TPN through the membrane. The C-terminus phenylalaninol is colored blue. PG lipid heads (orange), PE lipid heads (dark cyan), OL lipid chains (beige), TPN (green).

**Figure 4:**
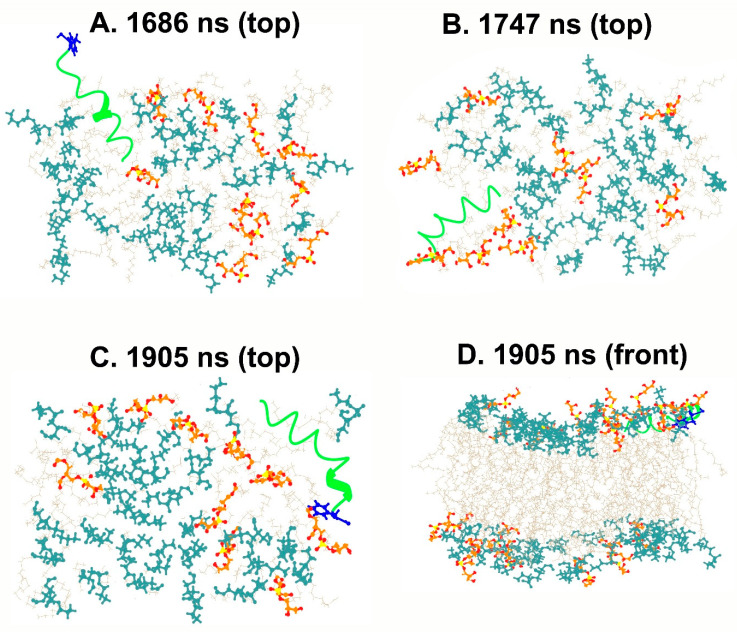
Snapshots from the end of the simulation at different time steps showing the movement of TPN through the membrane (top and front views). The aggregation of PE lipids is apparent in these images, while in the last graphic (**D**) we can observe membrane curvature. The C-terminus phenylalaninol is colored blue. TPN peptide can be observed to have evolved into its linear, spiral conformation reported by us in a previous report. PG lipid heads (orange), PE lipid heads (dark cyan), OL lipid chains (beige), TPN (green).

As discussed earlier, antimicrobial peptides may act on the bulk properties of the membrane, for example, affecting the curvature or aggregation of anionic lipids, or directly targeting specific lipids, which may cause serious defects in the structure and permeability of membranes. The TPN monopeptide was placed in the water environment above the bilayer membrane and simulated in multiple steps until 2.2 μs. The peptide seemed to move quickly from the aqueous phase to the bilayer surface within 40 ns and acquired an unfolded bent conformation. Soon after at 53 ns, the N-terminus entered the trans-membrane region as a folded spiral-shaped conformation. The C-terminal phenylalaninol remained outside and showed helical unfolding in the central region. At 300 ns, it is clear that the entire peptide is in a surface-bound state with its central region submerged and the characteristic spiral shape of TPN begins to form (Figure 3). The presence of the peptide introduces a curvature in the top layer and lipid clustering could also be observed. The anionic PG lipids surround the peptide, while zwitterionic PE lipids make a large aggregate. This displacement of lipid heads clearly introduces an empty space which is supposed to bring about disruption of the membrane. The same peptide conformation, lipid head arrangement, and empty space at the membrane surface can be observed throughout the simulation, for example at 1.5 μs. At around 1.6 μs, a linear, spiral conformation of the peptide begins to appear, and, finally, the previously reported native-like TPN XIIc conformation is obtained at 1.9 μs (Figure 4). This structure also introduces membrane curvature and aggregation of the surrounding anionic PG lipids.

### 3.2. Calculation of Lipid Order Parameters

**Figure 5:**
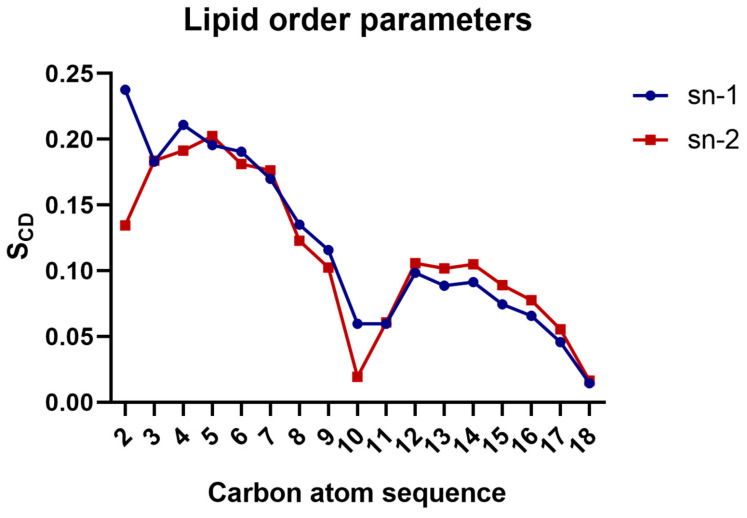
Average lipid order parameters calculated for the PE lipid chains.

The calculation of the lipid order parameters for the DOPE:DOPG membrane used in this study gave plateau values (carbon 4 to 6) for sn1 and sn2 averaging at 0.19 for both chains (Figure 5), which is slightly lower than the value of 0.21 for the pure DOPE membrane, as reported by Venable et al. [34]. However, the same parameter, when calculated for the same membrane composition but with an Alm F30/3 hexamer pore embedded in the transmembrane region, was even lower at 0.16 [35]. This shows that a single TPN peptide does not bring as much membrane disorder as observed in the presence of a transmembrane pore. Li et al. (2018) [36] conducted a study on a *Pseudomonas aeruginosa* mimicking membrane system, comprising DOPE and DOPG with a synthetic lipid and reported an average S_CD_ value for pure DOPE inner membrane of 0.180 and for DOPG of 0.112, while the same parameter calculated for this system has a value of 0.11, which shows slight disorder.

### 3.3. Conformational Dynamics of TPN during Interaction with a Bilayer Membrane

**Figure 6:**
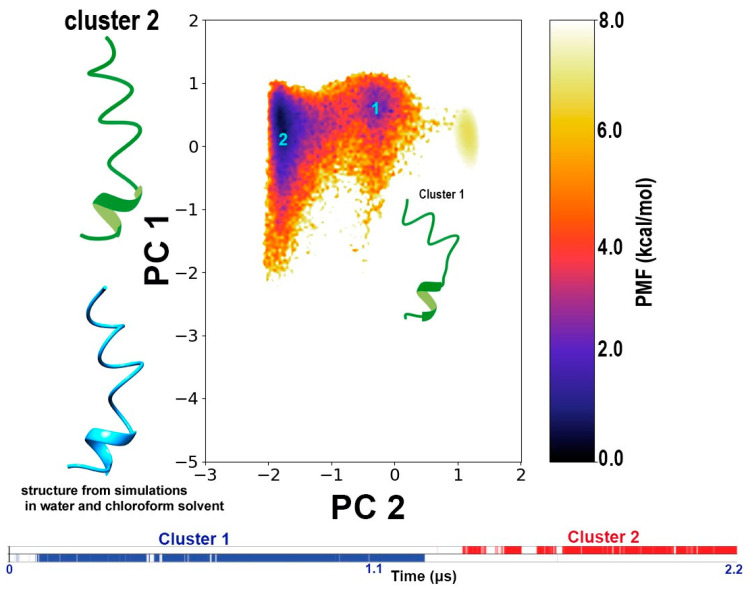
The dihedral angle-based PCA plot describing the two clusters obtained from these simulations regarding TPN structure evolution. The color bar represents the potential-of-mean force (PMF in kcal/mol) which represents the potential energy surface of the system. The curved structure representing cluster 1 lies at an unstable high energy level, while the other, linear coil-like structure representing cluster 2 forms at the true energy minimum. This indicates that the linear coiled structure is the most stable conformation of TPN within this membrane system. This structure was also obtained in our previous report on TPN simulation in water and chloroform [8]. The graphical representation of the simulation frames forming each cluster as a function of time shows that both conformations exist for a significant amount of time during this simulation.

In this work, no restraints were placed on peptide conformation and, therefore it was important to observe the structural changes in the peptide over the course of the simulation. We carried out a dihedral-based PCA to obtain the free energy landscape of the peptide structure observed during the interaction with the bilayer membrane. The PCs calculated based on the un-reweighted (boosted) trajectory resulted in two distinct minimum energy wells corresponding to two distinct conformations. However, when the free energy landscape is reweighted, only one of the conformation clusters appears in the energy minimum, while the other is observed at a very high energy. In other words, upon reweighting, i.e., removing the effect of boost energy from the simulation, the real potential energy surface revealed only one energetically stable conformation for TPN in a bilayer membrane. This conformation is a linear, coiled structure with a 3_10_ helix at the C-terminus, which was reported in our previous work as cluster 2 obtained from simulations in water and cluster 1 from simulations in chloroform [8] (Figure 6).

**Figure 7:**
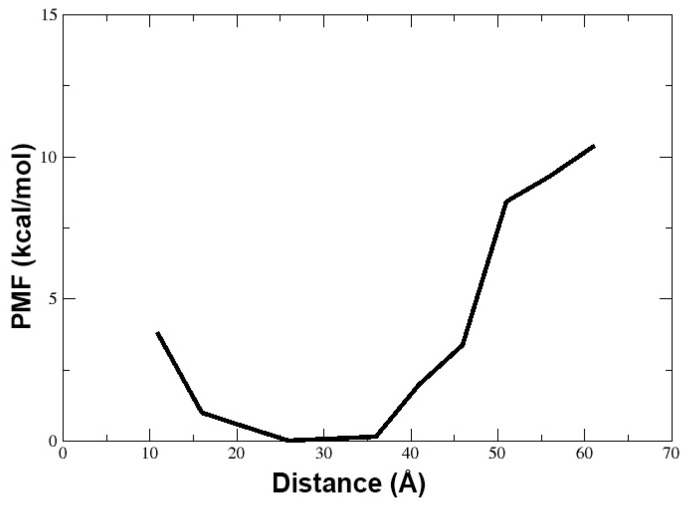
The reweighted PMF in kcal/mol for the distance between center-of-mass of the peptide and the center of membrane (in Å). The energy minimum was observed for a distance of 25–35 Å between the peptide and membrane center which signifies that the membrane bound conformation of the peptide is the most energetically stable state.

We also calculated the potential-of-mean-force (PMF in kcal/mol) by reweighting the distribution of the distance (in Å) calculated between the center-of-mass of the peptide and the center of the membrane (Figure 7). This distance includes the solved state of TPN in water to its journey to the hydrophobic core of the membrane. We observed that the energy minimum of this distance lies at 25 to 35 Å. It is known that the general width of the hydrophobic core of the membrane is 30–40 Å while the full bilayer thickness can vary from 75–100 Å. During our simulation, we observed that the TPN peptide remains bound to the surface containing lipid heads with some portions inserted within the hydrophobic region of the membrane. The PMF values also confirm that the most stable peptide configuration is found at a distance of 25 to 35 Å from the membrane center, which is the surface. The PMF rises sharply when the distance increases above 40 Å implying a high energy state of the peptide when above the membrane. If we compare these results with snapshots shown in Figure 4, we can say that the latter third of the simulation when the peptide has stabilized at the membrane surface without insertion and takes a linear form is its most stable state observed during this simulation.

## 4. Discussion

In this work, we placed a folded TPN at the water-bilayer interface to observe changes in the peptide structure and its insertion within the membrane. The study of bioactive peptides such as TPN is crucial to understanding their mechanism of action. We observed that the peptide remained bound to the membrane surface throughout the simulation and was stabilized via electrostatic interactions. There have been reports of amphipathic or hydrophilic short peptides binding and inserting within membranes and that behavior is coupled with their folding into an α-helix [37,38]. This simultaneous helix formation from unfolded conformation as the peptide binds to the membrane is due to the formation of hydrogen bonds between the amide groups [39]. There are contrary examples, like the pH-low insertion peptide (pHLIP), which binds in the unfolded form. In the case of TPN, we did not observe any increase in the α-helical content. The spiral-like conformation seems to be its native structure in equilibrium between the linear and bent backbone shapes. This conformation was also obtained during our previous simulations in water and chloroform. However, in water, this conformation is energetically stable, whereas in chloroform it is not. Therefore, despite reports of their poor solubility in water [40,41], peptaibols could be accurately modeled in a water solvent.

During the whole 2.2-μs-long simulation, the peptide does not attain a trans-membrane position across the width of the bilayer, which is an important step in forming ion channels. Multiple reasons can be attributed to this observation. It is known that peptaibol activity generally increases with the increase in their amount, indicating that a higher number of peptides may team up and form ion channels as a result. A single peptide may not be able to cross that barrier alone. Afanasyeva et al. [42] showed that the presence of another peptaibol, Alamethicin (Alm), could make free fatty acids in the membrane aggregate around the Alm molecule. Furthermore, Su et al. [43] studied a 1,2-di-O-phytanyl-sn-glycero-3-phosphocholine (DPhPC) bilayer and demonstrated that at potentials where the bilayer is stable, Alm assumes a surface-bound state with their helices at a large angle from the bilayer normal. Alm peptides are inserted when negative potentials are applied. We have not yet studied the effect of applied potential on TPN XIIc membrane insertion. An important computational work by Putzu et al. [44] on the behavior of Harzianin HK VI in DMPC membranes is also of relevance here. It is important to note that DMPC membranes are thinner than DOPE:DOPG membranes due to differences in lipid tail lengths. Therefore, a short peptide like Harzianin HK VI is more likely to attain a transmembrane, perpendicular to the bilayer plane conformation. Putzu et al. tested five peptide conformations: α-helix, 3_10_ helix, β-bend ribbon and fully extended. When the first three conformations are placed under restraint to maintain their secondary structures and starting orientation in the hydrophobic membrane region just below the lipid heads, all of them reorient with the peptide axis perpendicular to the bilayer plane. However, as the restraint on the 3_10_ helix and fully extended conformations are lifted, the peptide remains in the starting parallel conformation to the bilayer plane. It was found through NMR and circular dichroism experiments that the 3_10_ helix is the prominent conformation and its orientation parallel to the bilayer plane was found to be dominant. It becomes clear that even when algorithmically forced to attain a different transmembrane orientation, the most stable and dominant orientation remains at the membrane surface, parallel to the bilayer plane, just as we observed for TPN. Nevertheless, a single peptide seemed to bring significant membrane surface perturbation and points toward a carpet mechanism.

**Figure 8:**
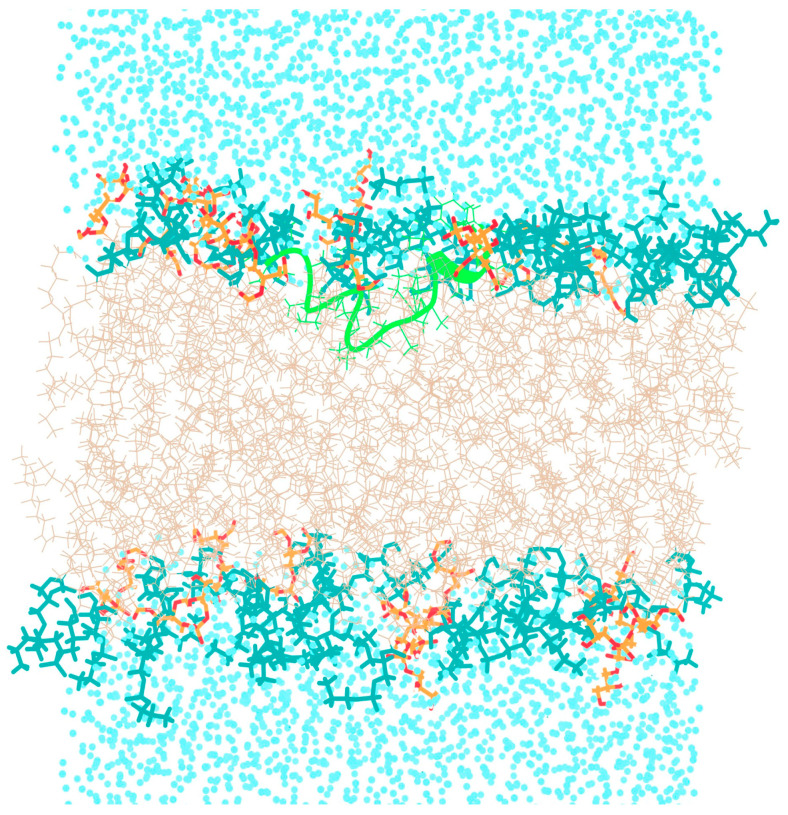
Snapshot of TPN immersed below the lipid heads except the terminal regions that interact with the lipid heads. The ‘nativecontact’ analysis recognized Ser2 and Gln17 to be mainly involved in this interaction. PG lipid heads (orange), PE lipid heads (dark cyan), OL lipid chains (beige), TPN (green).

We observed that TPN binding caused the clustering of lipids and a gap around the insertion area, which could indicate a carpet mechanism of action. Finger et al. [45] reported the clustering of PG lipids upon the binding of cyclic peptides. We also observed an increased disorder of the membrane, which is not surprising given that the peptide inserts well inside the transmembrane region. It would be beneficial to study the same system with an increased peptide-to-lipid ratio to observe their interaction and the possibility of forming pores across membranes. This was not the main goal of this study but we are in the process of setting up similar experiments. Interestingly, the N- and C-termini seem to prefer contact with the lipid heads, preventing complete insertion for a long time during the simulation. When we calculated native contacts between the peptide side chains and lipid headgroups, the Ser2 and Gln17 residues seemed to form contacts with the PG lipid head groups. Serine and glutamine are polar amino acids and are present at the two termini of the peptide chain. Therefore, we observed a long period in the trajectory when the peptide can be seen submerged below the lipid heads except at the termini that interact with the lipid heads (Figure 8). It should be noted that the TPN configuration is the same as cluster 1 in Figure 6, indicating that the ability of peptaibols to bend along their backbone is a crucial ability for successful membrane insertion.

The presence of helix-breaking residues like glycine and proline is also supported by the fact that they are almost always found in the central regions. Their presence may be required to allow the peptide chain to adopt bent conformations during the insertion process from the water-bilayer interface. In this study of the placement of a bioactive fungal peptaibol at the water-bilayer interface, we observed a number of effects including plausible lipid aggregation and membrane curvature. We also observed that two main conformations of the peptide could be obtained. These conformations were also observed during simulations in water and chloroform, where they were energetically stable in the former solvent but at a higher energy metastable state in the latter. This indicates that the folding simulations (using enhanced sampling methods) of peptaibols can be successfully carried out in water solvent.

## 5. Conclusions

By placing a folded conformation of a novel fungal peptaibol named TPN outside a simplistic representation of a bacterial bilayer membrane, we were able to observe the spontaneous peptide insertion from the water-bilayer interface. The insertion was accompanied by increasing membrane disorder and lipid clustering. The peptide entered the hydrophobic region of the membrane but always remained in the lateral position close to the surface, never reaching a trans-membrane position. Two main structural clusters were obtained: one with a bent, spiral-like conformation and the other with a linear, spiral-like conformation. The latter is the only energetically stable conformation according to free energy landscapes and lies in the true energy minimum. This conformation was also obtained as a stable structure during folding simulations in water in our previous work. Therefore, water solvent can be a suitable in silico environment to carry out folding simulations of peptaibols despite their poor solubility observed during in vitro experiments.

## Data Availability

Data are contained within the article.
